# Nitro-Oleic acid protects from neovascularization, oxidative stress, gliosis and neurodegeneration in oxygen-induced retinopathy

**DOI:** 10.1016/j.redox.2025.103634

**Published:** 2025-04-12

**Authors:** María Victoria Vaglienti, María Constanza Paz, Maria Victoria Gutierrez, Paula Virginia Subirada, Jose Luna, Gustavo Bonacci, María Cecilia Sánchez

**Affiliations:** a Departamento de Bioquímica Clínica, Facultad de Ciencias Químicas, Universidad Nacional de Córdoba, Córdoba, 5000, Argentina; b Centro de Investigaciones en Bioquímica Clínica e Inmunología (CIBICI), Consejo Nacional de Investigaciones Científicas y Técnicas (CONICET), Córdoba, 5000, Argentina; c Centro Privado de Ojos Romagosa S.A, Argentina

**Keywords:** Nitro-fatty acids, Retinal function, Proliferative retinopathies, Gliosis, IBA1+ retinal myeloid cells, Oxidative stress

## Abstract

Inflammation and oxidative stress are involved in Proliferative Retinopathies (PR). Müller glial cells (MGCs) and microglia play pivotal roles in pathological neovascularization (NV) and neurodegeneration in PR. Nitro-fatty acids are important electrophilic signaling mediators with anti-inflammatory and antioxidant properties. Herein, our goal was to evaluate the cytoprotective effect of nitro-oleic acid (NO_2_-OA) on neurons, MGCs and microglia in a mouse model of oxygen-induced retinopathy (OIR). NO_2_-OA induced vascular regrowth and reduced NV at P17 OIR, although no difference in the proangiogenic/antiangiogenic (VEGF-A/PEDF) balance was found between NO_2_-OA treatment and vehicle. In addition, Western blot and immunofluorescence assays showed that NO_2_-OA prevented gliosis at P17 OIR and decreased the number and activation of IBA1+ retinal myeloid cells. However, NO_2_-OA did not restore the decrease in expression of glutamine synthase (GS). Loss of retinal function in OIR mouse model measured by electroretinography was ameliorated, mainly at P26 OIR, after NO_2_-OA treatment. Western blot analysis of retinas from OIR mice revealed decreased levels of caspase-3 protein and increased number of TUNEL-positive cells at P26 compared to RA. Notably, these alterations were partially prevented after NO_2_-OA treatment. Besides, NO_2_-OA attenuates oxidative stress induced in MGCs exposed to aqueous humor from patients with different stages of PR. These findings highlight NO_2_-OA as a promising therapeutic strategy targeting both vascular and neuroglial components in PR, suggesting its potential clinical relevance.

## Highlights

• Inflammation and oxidative stress contribute to the pathological changes in retinopathy progression.
• Nitro-fatty acids stimulate transcriptional expression of Nrf2 downstream genes, NQO1 and SOD1, in retinas.
• Nitro-oleic acid ameliorates vascular alterations, glial stress and neurodegeneration involved in OIR mouse model.
• Nitro-oleic acid may have the potential to treat ROP, PDR and other neovascular and neurodegenerative eye diseases.

## Graphical abstract

**Figure:**
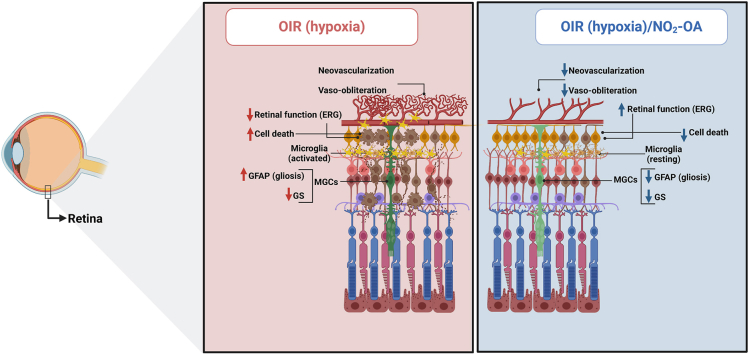

## 1 Introduction

Proliferative retinopathies (PR), such as Retinopathy of Prematurity (ROP) and Proliferative Diabetic Retinopathy (PDR), are major leading causes of blindness in pediatric and working-age adult populations, respectively [[1], [2], [3]]. Ischemic retinopathies are characterized by decreased oxygen (O_2_) supply to retinal tissue, which in advanced stages of the disease lead to ocular preretinal neovascularization (NV). Increasing evidence, from experimental models and clinical studies of PR, has provide insight and clarification to the mechanisms underlying vascular injury, leading to implementation of ocular anti-Vascular Endothelial Growth Factor (VEGF) therapies as the most prescribed treatment [4,5]. Other available treatments are laser photocoagulation, vitrectomy and cryotherapy [6]. Currently therapeutic approaches address late-onset NV. However, the clinical benefit conferred by these therapies is variable and many patients become refractory or resistant to treatment [7].

In addition to vascular changes, it is known that non-vascular cells such as glia and neurons are also affected, with presence of gliosis, IBA1+ retinal myeloid cells activation and progressive neurodegeneration characterized by retinal functional loss, altered neurotrophic balance and neuronal damage [[8], [9], [10], [11], [12]]. Indeed, Müller glial cells (MGCs) and microglial cells play a key role in the progression of pathological NV and neurodegeneration contributing to microvascular dysfunction [[13], [14], [15], [16], [17]]. This suggests that targeting both vascular and neural components concurrently could be an effective therapeutic approach for managing these complex diseases. In this sense, numerous evidences have shown that abnormal visual function, as well as decreased contrast sensitivity, precedes the detectable microvascular clinical signs in PR [18]. Thus, it is crucial the development of new drugs that not only can target NV, but can also protect neuron and glial cells from degeneration. In this regard, the mouse model of oxygen-induced retinopathy (OIR) is a powerful tool for elucidating the mechanisms involved in PR and developing new therapeutic strategies [19]. The *in vivo* OIR model has shown retinal vaso-obliteration and NV, with abnormal neuronal function as consequence of loss of ganglion, bipolar, amacrine, and photoreceptors cells. In addition, gliosis and increase in number and activation of IBA cells are typical features of PR [8,9,20,21].

In this context, an unexplored therapeutic strategy in retina is the modulation of transcription factors involved in antioxidant defense, such as the nuclear factor erythroid 2-related factor 2 (Nrf2), which regulates the expression of phase II antioxidant enzymes such as heme oxygenase-1 (HO-1), NAD(P)H quinone oxidoreductase 1 (NQO1), as well as enzymes involved in glutathione biosynthesis, among others. In the retina, the protective effect of Nrf2 has been described in both ROP and DR [[22], [23], [24]]. Indeed, Lutty et al. and Wei et al. have highlighted the importance of Nrf2, showing that Nrf2 knockout mice (Nrf2^−/−^ KO) in the OIR model present an increase in the avascular area and NV [24,25]. Furthermore, the administration of pharmacological Nrf2 activators, dh404 and RS9, decreased the avascular and neovascular areas and permeability of retinal blood vessels [26,27]. Additionally, *in vitro* and *in vivo* studies demonstrated that Nrf2 regulates tip cell formation and vascular branching on the endothelial cells (ECs), inducing reparative angiogenesis in the tissues [13,23].

The nitroalkenes are the product of the reaction between nitric oxide-derived species and unsaturated fatty acids, forming a group of endogenous electrophilic lipid mediators with a variety of anti-inflammatory and antioxidant properties [28,29]. These nitro-fatty acids (NO_2_-FA) are mainly represented in plasma and urine of healthy individuals by conjugated nitro-linoleic acid (NO_2_-CLA). Nitration of fatty acid occurs, predominantly, in the gastrointestinal tract, and in lesser extension at inflammatory sites or during ischemic events to then reach systemic distribution. Although NO_2_-CLA is endogenous generated, NO_2_-OA is the pharmacological candidate to be administrated in experimental model due to high stability and tolerance [30]. In the last few years, numerous studies have demonstrated their anti-inflammatory and cytoprotective effects [31]. These actions are mediated through Michael adduction reaction of NO_2_-FA with key cellular proteins in the Keap1/Nrf2 antioxidant pathway, and the proinflammatory NF-kB and TLR4 signaling pathways, in inflammation, ischemia/reperfusion-induced cardiac injury, and diabetic experimental models [32,33].

Given that the induction of the retinal antioxidant defense system through the activation of the Keap1/Nrf2 signaling pathway is a protective mechanism in PR [22,[25], [26], [27]], we hypothesized that NO_2_-OA could exert beneficial actions on the pathological alterations of OIR such as retinal vaso-obliteration and NV, neuronal damage, loss of retinal function, gliosis and IBA1+ retinal myeloid cells activation.

In this study, the murine model of OIR was used to evaluate the effect of NO_2_-OA on the vascular and nonvascular abnormalities. Furthermore, we also evaluated the protective role of NO_2_-OA against oxidative stress induced in MGCs by aqueous humor from patients with PR. These findings would provide insights with the purpose to identifying new therapies to fully rescue of the RP and prevent visual deficits.

## 2 Materials and methods

**Materials:** A spontaneously immortalized human Müller glial cell line (MIO-M1) was kindly provided by G. Astrid Limb (UCL Institute of Ophthalmology and Moorfields Eye Hospital, London, UK). Cell culture plates were purchased from Greiner Bio-One (Frickenhausen, Germany). Nitro-oleic acid (NO_2_-OA) was synthesized as previously described [34,35] and was generously donated by Bruce A. Freeman and Francisco Schopfer (University of Pittsburgh, Pittsburgh, PA).

### 2.1 Animals

WT C57BL/6J mice were handled according to guidelines of the ARVO Statement for the Use of Animals in Ophthalmic and Vision Research. Experimental procedures were designed and approved by the Institutional Animal Care and Use Committee (CICUAL) of the School of Chemical Sciences, National University of Córdoba (RD-2022-1731-E-UNC- ME#FCQ). All efforts were made to reduce the number of animals used.

### 2.2 Oxygen-induced retinopathy (OIR) mouse model

In a model of OIR [36], litters of mice pups with their nursing mothers were exposed in an infant incubator to high oxygen concentration (75 % ± 2 % O_2_, hyperoxia period), during 5 days from postnatal (P) day 7 to P12. Oxygen was checked twice daily with an oxygen analyzer (Teledyne Analytical Instruments, CA, USA). Next, mice were housed in room air (RA) for an additional time period of 5 or 14 days (relative hypoxic period, P12–P17 or P12–P26). Age-matched control C57BL/6 mice were exposed continuously to RA (normoxic conditions). Animals were maintained in clear plastic cages with standard light cycles (12 h light/12 h dark). At P12, some OIR mice were intraocular (i.o.) injected with 1 μl of NO_2_-OA (50 μM) in DMSO 10 %: PEG400 10 %: PBS 80 % or vehicle (DMSO 10 %: PEG 400 10 %: PBS 80 %) used as controls. The NO_2_-OA final concentration (5 μM) was obtained according to previous *in vitro* studies [13] considering also the 10 μl of vitreous volume (1/10 dilution). Briefly, P12 wild-type mice were fully anesthetized using isoflurane inhalation and monitored throughout the procedure. In addition, pups were topically anesthetized with one drop of proparacaine hydrochloride 0.5 % (Anestalcon, Alcon), and exophthalmia was induced with one drop of tropicamide 1 % (Midril, Alcon, Buenos Aires, Argentina). Intravitreal injections were performed under an operating stereo microscope (Nikon SMZ645, Microlat, Córdoba, Argentina) to ensure precision and eyes were punctured at the upper nasal limbus as previously [37]. Correct injection placement was confirmed by observing the reflux of a small fluid bubble at the injection site. Then, intraperitoneal (i.p.) injection reinforcement on days P14, P17, P20 and P23 with NO_2_-OA (15 mg/kg) in PEG 400: PBS was done. Control mice were injected on the same days with vehicle (PEG400: PBS). Mice were sacrificed at two typical time points in the OIR mouse model: P17 (peak of NV) and P26 (vascular alteration resolution) [8,9]. Eyes or retinas of sacrificed mice were collected and processed for Western blot, real-time PCR (qRT-PCR), *flat-mount*, immunohistochemistry, or immunofluorescence. At least six mice per group were used for each condition in the survival times examined. Data were collected from both males and females and the results were combined as there were no apparent sex differences.

### 2.3 Ocular compatibility/tolerance assay in wild type C57BL/6 mice

Adults wild type C57BL/6J mice exposed to standard light cycles (12 h light/12 h dark) were i.o. injected with 1 μl of NO_2_-OA (50 μM) in DMSO 10 %: PEG400 10 %: PBS 80 %, vehicle (DMSO 10 %: PEG400 10 %: PBS 80 %) or PBS during the light cycle (day 0). Animals were maintained in clear plastic cages with standard light and i.p. injected with NO_2_-OA (15 mg/kg) in PEG 400: PBS, vehicle (PEG 400: PBS) or PBS every 2 days (days 2, 5, 8, 11 and 14). Retinal electrophysiological studies were performed at 3, 7 and 15 days after i.o. injection, followed by euthanasia of animals for histopathological evaluation.

### 2.4 Electroretinography (ERG)

Scotopic electroretinography was assessed at P17 and P26 RA and OIR mice with or without NO_2_-OA treatment, as previously described [9,38]. Briefly, after overnight (ON) dark adaptation and under dim red illumination, mice were anesthetized via i.p. with ketamine (90 mg/kg)/xylazine (8 mg/kg), the pupils were dilated with 1 % tropicamide and the cornea was lubricated with gel drops of 0.4 % polyethylene glycol 400 and 0.3 % propylene glycol (Systane, Alcon, Buenos Aires, Argentina) to prevent damage. Mice were exposed to stimuli at a distance of 20 cm. A reference electrode was inserted on the back between the ears, a grounding electrode was attached to the tail, and a gold electrode was placed in contact with the central cornea. Electroretinograms were simultaneously recorded from both eyes and ten responses to flashes of unattenuated white light (10 cd s/m^2^, 0.2 Hz) from a photic stimulator (light-emitting diodes) set at maximum brightness were amplified, filtered (1.5-Hz low-pass filter, 1000 high-pass filter, notch activated) and averaged (Akonic BIO-PC, Argentina). The a-wave was measured as the difference in amplitude between the recording at onset and trough of the negative deflection, and the b-wave amplitude was measured from the trough of the a-wave to the peak of the b-wave. The latencies of the a- and b-waves were measured from the time of flash presentation to the trough of the a-wave or the peak of the b-wave, respectively. Responses were averaged across the two eyes for each mouse.

### 2.5 Labeling of flat-mount retinas

Mice were euthanized at P17 and eyes were enucleated and fixed with freshly prepared 4 % paraformaldehyde (PFA) for 1–2 h at room temperature (RT). Corneas were removed with scissors along the limbus and the whole retinas were dissected. Then, they were blocked and permeabilized in tris-buffered saline (TBS) containing 5 % Bovine Serum Albumin (BSA) and 0.1 % Triton-X-100 during 6 h at 4 °C. After that, retinas were incubated ON with 0.02 μg/μL of Isolectin IB4 Alexa fluor-488 conjugate (GSA-IB4) from Molecular Probes, Inc. (Eugene, OR, USA). Then, retinas were washed with TBS containing 0.1 % Triton-X-100, stored in PBS at 4 °C and examined by confocal laser-scanning microscopy (Olympus FluoView FV1200; Olympus Corp., New York, NY, USA). Vaso-obliteration, NV and total retinal area were measured using FIJI (National Institutes of Health, Bethesda, MD, USA) [39].

### 2.6 Retinal cryosection, protein extract and RNA sample preparation

For cryosection, eyes were enucleated, fixed during 1–2 h with 4 % PFA at RT, and incubated ON in a sucrose gradient (10 %, 20 %, and 30 %) for at least 1 day at each concentration in PBS at 4 °C. Then, they were embedded in optimum cutting temperature (OCT) (Tissue-TEK, Sakura) compound, and 10 μm thick radial sections were obtained by using a cryostat, according to general methods as described [40]. Retinal cryosections were then stored at −20 °C under dry conditions until immunohistochemical analysis. Neural retinas were dissected from RPE/choroid layers for Western blot and qRT-PCR analysis. Protein extracts were obtained from retinas after homogenization with lysis buffer containing 20 mM Tris-HCl pH 7.5, 137 mM NaCl, 2 mM EDTA pH 8, 1 %, Nonidet P40, 1 mM phenylmethylsulfonyl fluoride (PMSF), 2 mM sodium orthovanadate and protease inhibitor cocktail (Sigma Aldrich, St. Louis, MO) [40], and were sonicated during 20 s at 40 % amplitude. In addition, some neural retinas were disrupted in Trizol (500 μL) (Invitrogen) and were stored at −80 °C until RNA extraction. All the assays were performed in triplicate and results are representative of at least three independent experiments.

### 2.7 Immunofluorescence labeling in cryosections

For detection of GFAP, IBA-1 and CD31 (PECAM-1) in immunofluorescence, retinal cryosections were washed with PBS, blocked with 2 % of BSA in PBS containing 0.2 % tween 20 (T20), for 1 h and then incubated ON at 4 °C with the following primary antibodies: rabbit polyclonal anti-GFAP (1/100; Dako, Carpinteria, CA), rabbit polyclonal anti-IBA-1 (1/250; Abcam Inc., Cambridge, MA) and mouse monoclonal anti-CD31 (1/50; Abcam Inc., Cambridge, MA). Then, sections were washed with PBS 0.2 % T20 and incubated with secondary antibodies including goat against rabbit or mouse IgG conjugated with Alexa Fluor 488 and 594 (1/800; Molecular Probes, Eugene, OR, USA) respectively, during 1 h at RT. The sections were also counterstained with Hoechst 33258 (1/3000; Molecular Probes) for 7 min. After a thorough rinse, the sections were mounted with Fluor Save (Calbiochem, La Jolla, CA) and cover slipped. The labeling was visualized using a confocal laser-scanning microscope (Olympus Fluvial FV300 or FV1200; Olympus Corp., New York, NY, USA) or PhenoImager Fusion microscope (Akoya Biosciences and The Spatial Biology Company; Marlborough, MA 01752, USA). Finally, images were processed with microscope software FIJI (National Institutes of Health, Bethesda, MD, USA) [39] or InForm® with MOTiF™ software. Negative controls without incubation with primary antibody were carried out to avoid unspecific staining (data not shown).

### 2.8 Evaluation of retinal structure in cryosections

Retinal cryosections were stained with hematoxylin and eosin (H&Eo), with the purpose to assess the integrity of the retinal layers. Using bright field microscopy (Nikon eclipse TE 2000), cryosections of mouse retinas were examined and the thickness of the retinal layers was measured using NIS ELEMNTS software.

### 2.9 Western blot

Protein concentration of retinal extracts was determined by BCA kit (Pierce, Buenos Aires, Argentina) and 20–25 μg of proteins were electrophoresed in 15 % SDS-PAGE. After electrophoresis, proteins were transferred to nitrocellulose membranes (Amersham Hybond ECL; GE Healthcare Bio-Sciences AB, Uppsala, Sweden). To prevent nonspecific binding, membranes were blocked with 5 % milk in TBS containing 0.1 % T20 (TBST) during at least 1 h at RT. Then, blots were incubated with primary antibodies in 5 % BSA TBST ON at 4 °C. The following antibodies were used: rabbit polyclonal anti-GFAP (1/1000; Dako, Carpinteria, CA), mouse monoclonal anti-GS (1/2000; Millipore Corporation MA, USA), rabbit polyclonal anti-caspase 3 (1/1000; Sigma-Aldrich), mouse polyclonal anti-alpha tubulin (1/1000; Abcam Inc., Cambridge, MA) and mouse monoclonal anti-βactin (1/2000; Sigma-Aldrich). Blots were incubated with IRDye 800 CW donkey anti-rabbit Ig or IRDye 700 CW donkey anti-mouse IgG antibodies (1/15000 in 5 % BSA TBST) for 1 h, protected from light. After washing with TBST, membranes were visualized and quantified using the Odyssey Infrared Imaging System (LI-COR, Inc., Lincoln, NE, USA).

### 2.10 qRT-PCR

Total RNA was extracted from neural retinas using Trizol (Invitrogen), according to the manufacturer's instructions and was processed as previously reported [41]. Briefly, 1 μg of total RNA was reverse-transcribed in a total volume of 20 μL using random primers (Invitrogen, Buenos Aires, Argentina) and 50 U of M-MLV reverse transcriptase (Invitrogen, Buenos Aires, Argentina). For qPCR, cDNA was mixed with 1x SYBR Green PCR Master Mix (Applied Biosystems) and the forward and reverse primers (NQO-1 forward: AGGATGGGAGGTACTCGAATC/NQO-1 reverse: AGGCGTCCTTCCTTATATGCTA; SOD1 forward: AACCAGTTGTGTTGTCAGGAC/SOD1 reverse: CCACCATGTTTCTTAGAGTGAGG; VEGF-A forward: GGAGACTCTTCGAGGAGCACTT/VEGF-A reverse: GGCGATTTAGCAGCAGATATAAGAA; PEDF forward: TCGAAAGCAGCCCTGTGTT/PEDF reverse: AATCACCCGACTTCAGCAAGA). qPCR was carried out on an Applied Biosystems 7500 Real-Time PCR System with Sequence Detection Software v1.4. The cycling conditions included a hot start at 95 °C for 10 min, followed by 40 cycles at 95 °C for 15 s and 60 °C for 1 min. Specificity was verified by melting curve analysis. Results were normalized to GAPDH (forward: CAGAACATCATCCCTGCAT/reverse: GTTCAGCTCTGGGATGACCTT). Relative gene expression was calculated according to the 2-ΔΔCt method. Each sample was analyzed in triplicate. No amplification was observed in PCRs using as template water or RNA samples incubated without reverse transcriptase during the cDNA synthesis (data not shown).

### 2.11 Quantification IBA1+ retinal myeloid cells

IBA1+ retinal myeloid cells were quantified in retinal cross sections immunostained with the Iba1 antibody (Abcam Inc., Cambridge, MA) using InForm® with MOTiF™ software and the average values from separate sections/eye crossing the optic nerve were combined to produce a mean value. Images were obtained with Akoya Biosciences- Phenoimager Fusion microscope. We counted the number of total IBA1+ retinal myeloid cells. Additionally, activated IBA1+ retinal myeloid cells were identified based on morphology [42] and defined as IBA1+ cells with a large soma size and no extending processes.

### 2.12 TUNEL assay

Cell death was examined by terminal deoxynucleotidyl transferase biotin dUTP nick end labeling (TUNEL) assay (Roche, Mannheim, Germany) following the manufacturer's instructions. Slides were counterstained with methyl green to visualize retinal layers and then mounted with DPX Mounting Media (Sigma-Aldrich, St. Luis, MO). Negative controls without enzyme were processed in order to avoid false positive results (data not shown). For each section, TUNEL-positive nuclei staining brown were counted from all the retina using InForm® with MOTiF™ software and the average values from separate sections/eye crossing the optic nerve were combined to produce a mean value. Images were obtained with Akoya Biosciences- Phenoimager Fusion microscope.

### 2.13 Cell line and culture reagents

MIO-M1 cells were grown in complete high-glucose Dulbecco's modified Eagle's medium (DMEM; Invitrogen, Buenos Aires, Argentina) containing 10 % fetal bovine serum (FBS), 2 mM l-glutamine (GlutaMAX; Invitrogen), and 50 U/mL penicillin/streptomycin (Invitrogen). Cells were maintained in a 5 % CO_2_ humidified culture incubator at 37 °C.

### 2.14 Dichlorofluorescein assay

The reactive oxygen species (ROS) production in MIO-M1 cells was quantified using the dichlorofluorescein (DCF) assay, following a previously described procedure [43]. Briefly, MIO-M1 cells were treated with NO_2_-OA (5 μM) or vehicle (0.1 % v/v methanol) for 2 h before adding patients’ aqueous humor for 3 h. A 20,70-dichlorofluorescein diacetate probe (5 μM) was added, and the cells were incubated for 30 min. The DCF fluorescence intensity was measured with an FACSCanto II flow cytometer (BD Biosciences) and analyzed with FlowJo software (ThreeStar, FlowJo eI_V10).

### 2.15 Patient samples

The protocol was approved by Ethics Committee in Health Research (CIEIS), Prof. Dr. Marcelino Rusculleda (Córdoba, Argentina), and the study was conducted according to standards of the Helsinki Declaration. Written informed consent was obtained from all participants before their enrollment. Patients older than 21 years old with type-2 diabetes mellitus were recruited between September 2023 and January 2024 from Centro Privado de Ojos Romagosa- (Córdoba, Argentina). Type 2 diabetes mellitus was diagnosed according to the American Diabetes Association (2022) criteria [44]. Patients were defined as a self-reported previous history of physician-diagnosed type 2 diabetes mellitus treated with insulin or oral hypoglycemic agents and those having a diagnosis of diabetes of at least 5 years were selected. The duration of the disease was defined as the interval between the first diagnosis of diabetes and the time of enrollment in the present study. A questionnaire was conducted to obtain basic information (age & sex), and additional data (including the use of insulin and oral hypoglycemic therapy and any history of other systemic diseases). A complete ophthalmological examination included an external examination of the eye and adnexa (including conjunctiva, sclera and lid position or abnormalities), pupil responsiveness (including but not limited to abnormal pupil shape, unequal pupils, abnormal reaction to light or afferent pupillary defect), slit-lamp biomicroscopic examination (cornea, anterior chamber, iris, and lens). Intraocular pressure measurements performed using a Goldmann applanation tonometer performed prior to pupil dilation. The examination also included a dilated indirect ophthalmoscopic examination (including evaluation of the posterior segment and vitreous, optic nerve, retinal vasculature, peripheral retina, and macula) conducted by one of the 2 retinal specialist and dilated fundus photography was done after the complete examination. Seven fields of 30° color fundus photographs with stereoscopic images of the optic disc and macula were taken through the dilated pupils of each patient, using a digital fundus camera (Zeiss Visucam Pro; Oberkochen, Germany). Two retinal specialist ophthalmologists determined the presence and classification of PDR in a masked manner. All PDR and neovascular glaucoma patients had, during the last 2 months prior to surgery, different degrees of active neovascularization (disk, arcade or neovascular glaucoma) with or without tractional retinal detachment. Anterior chamber taps were performed under topical anesthesia by microscopic observation of the eye with the patient lying down under a surgical microscope. All this procedure was taken before an i.o. injection of an antiangiogenic drug has been applied. By contrast, control group samples were taken before cataract surgery. A drop of 5 % povidone iodine was applied to the eye and a limbal paracentesis was performed with a sterile tuberculin syringe with a 30-gauge needle. It was inserted into the anterior chamber, and 0.2 ml of aqueous was removed and stored frozen at −80 °C until assayed. Three samples were collected from diabetic patients with PDR, three samples from the patients with neovascular glaucoma, and three samples from non-diabetic patients undergoing cataract were used as a control group as was previously described [45]. Patients with a vitreous hemorrhage were not included in this study.

### 2.16 Statistical analysis

Statistical analysis was performed using the GraphPad Prism for the statistical analysis, the GraphPad Prism 8.0 program was used. A p-value <0.05 was considered statistically significant. Parametric studies were selected given the homogeneity of variances evaluated by the F or Barlett test. Two-tailed unpaired t or Mann Whitney tests were used in analysis of two groups. One-way analysis of variance (ANOVA) followed by post-test of Dunnett's multiple comparison (comparison with control) or Tukey's (comparison between all groups) were used to determine the statistical differences between more than 2 different groups. Two-way ANOVA followed by Bonferroni post-test was used in comparisons between groups when two variables were affecting the dependent variable. Mean ± standard error (SEM) is shown in graphs analyzed with parametric tests.

## 3 Results

### 3.1 Characterization of NO_2_-OA actions on Nrf2 downstream genes in retina from RA mice

**Fig. 1:**
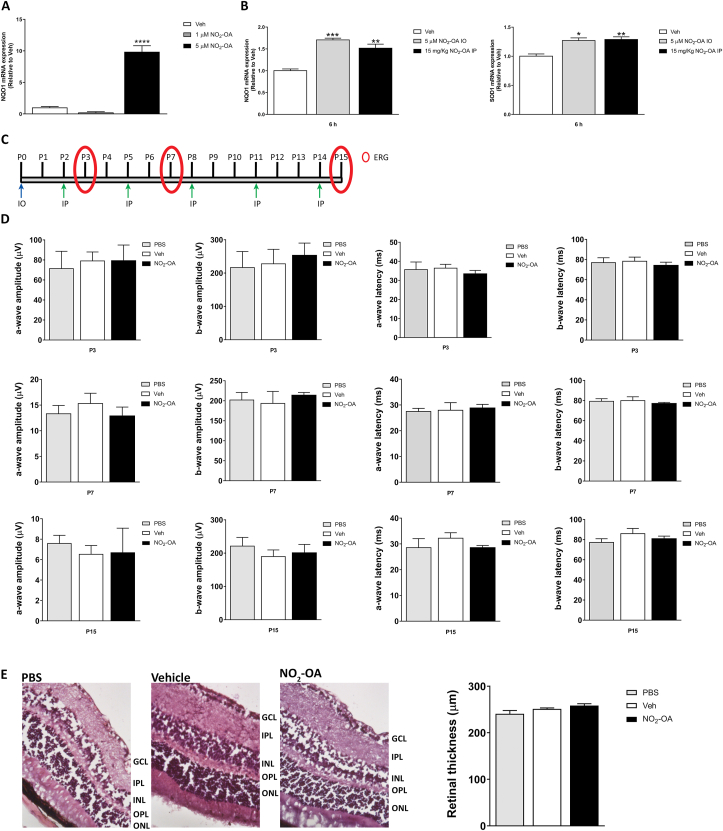
NO_2_-OA actions on Nrf2 downstream genes and NO_2_-OA cytotoxicity in healthy retina from RA mice. **A)** NQO1 mRNA was quantified by qRT-PCR in retinas treated for 6 h with NO_2_-OA (1 or 5 μM) or vehicle (control) in adult's mice. Results were normalized to GAPDH and expressed according to the 2-ΔΔCt method using as calibrator the mRNA level obtained from vehicle mice retinas. **B)** NQO1 and SOD1 mRNA was quantified by qRT-PCR in neurosensory retinas at P12 treated (i.o. or i.p.) for 6 h with NO_2_-OA (5 μM) or vehicle (control). Results were normalized to GAPDH and expressed according to the 2-ΔΔCt method using as calibrator the mRNA level obtained from P12 vehicle mice retinas. **C)** Experimental Scheme indicates the days of injection and time points for ERG. **D)** Amplitudes and latencies of a- and b-waves from scotopic ERG were recorded at P3, P7 and P15 following the injection scheme described above. i.o. injection: NO_2_-OA (5 μM), Vehicle, or PBS, and i.p.: NO_2_-OA (15 mg/kg), PBS, or Vehicle in adult mice. Data show the average of responses over both eyes, in four mice per condition. **E)** Representative histological Hematoxylin and Eosin staining sections of the retina at P15. The graph represents the retinal thickness (μm) at P15, in four mice per condition. Data are presented as mean ± SEM. Data were analyzed by one-way ANOVA followed by Tukey's post-test. ∗p < 0.05, ∗∗p < 0.01, ∗∗∗p < 0.001, ∗∗∗∗p < 0.0001. **D** and **E**) No statistically significant differences were found in the evaluated conditions.

We have, recently, shown the beneficial and protective effects of NO_2_-OA in modulate oxidative stress by activating the Keap1/Nrf2 antioxidant pathway in MGCs [13]. Thereby, we want to evaluate NO_2_-OA action on the expression of Nrf2 downstream target gene, such as NQO1. For this purpose, qRT-PCR of the whole retina from adult mice i.o. injected with NO_2_-OA (final concentration of 5 μM) showed a significantly increased after 6 h in NQO1 expression with respect to vehicle control. We have, also, tested lower concentration of NO_2_-OA (1 μM) but not changes in NQO1 retinal expression were observed (Fig. 1A). Next, we evaluated the effect of NO_2_-OA in retinas from mouse pups at P12 RA. In this experimental setting, NO_2_-OA significantly increased NQO1 and SOD1 transcriptional expression compared to control retinas (Fig. 1B). When we evaluate if systemic administration of NO_2_-OA (15 mg/kg) affect genes expression in retina, results similar to those of i.o. administration was observed (Fig. 1B). Therefore, these results demonstrate that NO_2_-OA induces the transcriptional expression of antioxidants enzymes in RA retinas from P12 pups and adult mice.

Then, we evaluated whether 5 μM of NO_2_-OA induced cytotoxicity by disturbing the functional status of retinas and structure by electroretinography (ERG) and histology, respectively. Adult C57BL/6 wild-type mice were i.o. injected with one dose of NO_2_-OA (final concentration of 5 μM) on day 0, and then received i.p. injections with NO_2_-OA (15 mg/kg) every two days (booster doses on days 2, 5, 8, 11, and 14) (Fig. 1C). A control mice groups were injected with vehicle and PBS. NO_2_-OA injected group did not show significant differences in a- and b-waves amplitude and latency of ERG compared to vehicle and PBS at days 3, 7 and 15 (Fig. 1D). Moreover, histological analysis in retinal thickness at day 15 did not show significant change in mice treated with NO_2_-OA or vehicle compared to control group (PBS) (Fig. 1E). These results indicate that retinal function and histology are not affected after administration of NO_2_-OA or vehicle at the doses and time evaluated in adult mice.

### 3.2 NO_2_-OA promotes reparative angiogenesis without modify VEGF-A expression in OIR mice

**Fig. 2:**
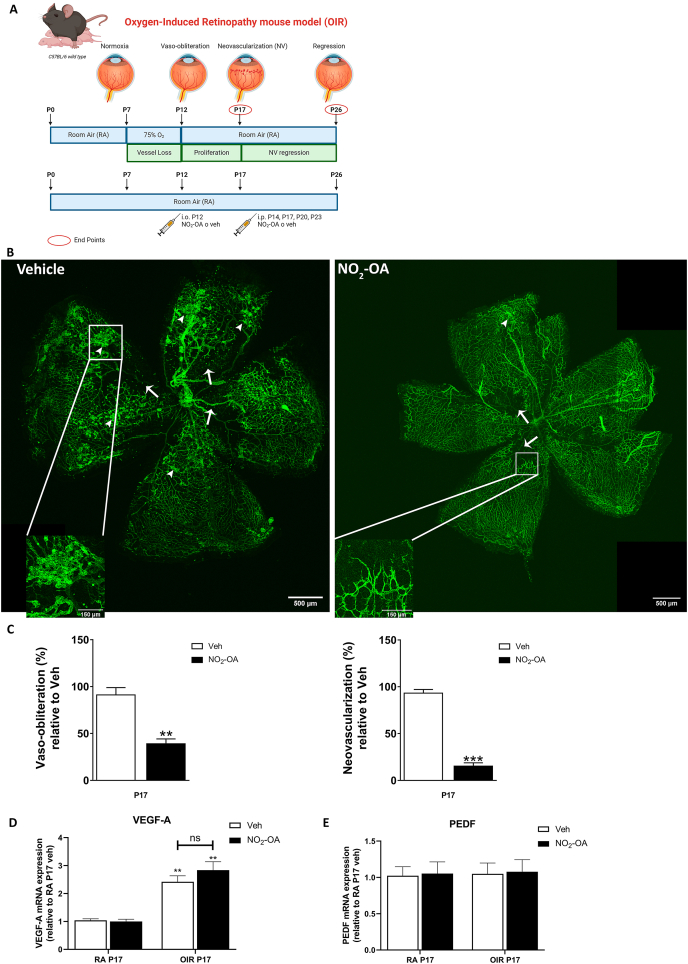
NO_2_-OA promotes reparative angiogenesis without modify VEGF-A expression in OIR. **A)** Scheme representative of the OIR treatment and the time points of NO_2_-OA or vehicle injections during development of the experimental mouse model. Bio render free version of the software was used. **B)** Representative images of whole mount retinas at P17 stained with the vascular marker GSA-IB4 in vehicle or NO_2_-OA (5 μM)-injected eyes. Arrows indicate avascular area and arrowhead indicate NV area. **C)** The VO (%) was quantified as the ratio of central avascular area to whole retinal area and the NV (%) was quantified as a percentage of whole retinal area. Data are presented as mean ± SEM. Data were analyzed by *t*-test. ∗∗p < 0.01, ∗∗∗p < 0.01. **D-E)** VEGF-A and PEDF mRNA was quantified by qRT-PCR in neurosensory retinas of P17. Results were normalized to GAPDH and expressed according to the 2-ΔΔCt method using as calibrator the mRNA level obtained from P17 RA vehicle mouse retinas. Data are presented as mean ± SEM. Data were analyzed by two-way ANOVA followed by Bonferroni's post-test. ns, non-significant, ∗∗p < 0.01.

Previous works have demonstrated that animals with genetic ablation of Nrf2 (Nrf2^−/−^) in the OIR model significantly increased the avascular area at P17, accompanied by a considerable exacerbation in pathologic preretinal NV in comparison with wild type mice [[23], [24], [25]]. Hence, we evaluated whether NO_2_-OA could prevent vascular alterations in OIR mice model (Fig. 2A). NO_2_-OA treatment, showed an increase in the revascularization of the central retina or reparative angiogenesis and a decrease in neovascular area in *flat-mount* retina labeled with GSA-IB4 lectin compared to control vehicle at P17 OIR (Fig. 2B). Quantitative analysis revealed that the avascular and neovascular area was reduced significantly after NO_2_-OA's treatment with respect to control vehicle (Fig. 2C). These results suggest that NO_2_-OA promotes a functional reparative angiogenesis in the ischemic retina.

On the other hand, retinal ischemia stimulates the secretion of several growth factors, mainly VEGF, which is an important mediator of vascular permeability and pathologic NV in the eye, leading to partial or total loss of vision [4,9]. Interestingly, it has been suggested that oxidative stress may cause pathologic features either independently or in association with VEGF signaling in retinopathies [46]. Since we demonstrated that treatment with NO_2_-OA reduces avascular and neovascular area in the OIR mouse model, then we analyzed whether this effect was mediated by regulation in VEGF levels. As we expected, retinas from OIR mice revealed a significant increase in VEGF-A mRNA levels at P17 with respect to RA (Fig. 2D) evaluated by qRT-PCR. NO_2_-OA treatment was unable to regulate the expression of VEGF-A mRNA levels induced by hypoxia in the OIR model. In addition, the balance in the levels of PEDF and the proangiogenic VEGF-A is perturbed in a range of retinal neovascular diseases. PEDF is antiangiogenic and can inhibit the growth of blood vessels in the eye induced in a variety of ways [47,48]. Thus, we have analyzed the PEDF mRNA expression in RA and OIR mice injected with NO_2_-OA or vehicle at P17. NO_2_-OA did not affect PEDF mRNA levels in OIR P17 mice compared to vehicle (Fig. 2E). Thus, our results suggest that NO_2_-OA did not modulate the proangiogenic/antiangiogenic (VEGF-A/PEDF) balance in our OIR model.

### 3.3 NO_2_-OA attenuates gliosis in retinas at P17 OIR mice

**Fig. 3:**
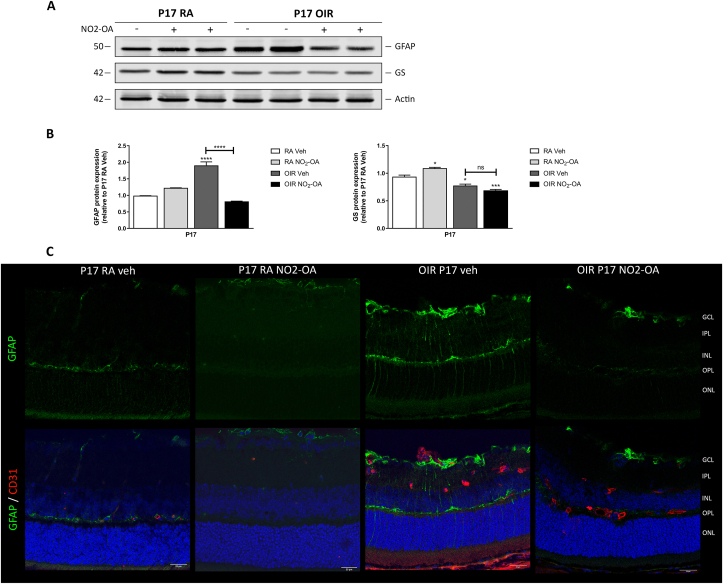
NO_2_-OA prevents gliosis in retinas at P17 OIR. **A)** Representative Western blot of GFAP and GS from retinas of RA and OIR mice injected or not with NO_2_-OA at P17. β-Actin is shown as a loading control. **B)** Quantification intensity of GS and GFAP was carried out by densitometry and then, normalized to β-Actin. Data are presented as mean ± SEM. ns, non-significant, ∗p < 0.05, ∗∗∗p < 0.001, ∗∗∗∗p < 0.0001. **C)** Representative immunofluorescence analysis of GFAP (green) in cryosections of RA and OIR mouse retinas at P17 (upper panels). Double labeling using an astrocyte and activated MGC marker, GFAP (green) in combination with an EC marker, CD31 (red) (lower panels). Cell nuclei were counterstained with Hoechst 33258 (blue). Scale bar: 25 μm.

It has been described that glial activation in retina, is a protective response to initial injury, but if the stressor persists may be a sign of bad prognosis. In previous works, we have shown that retinas exhibited neuroglial lesions and loss of retinal function, in addition to neovascular proliferation changes at P17 OIR [8,9]. Therefore, we evaluated whether NO_2_-OA could attenuate gliosis response at P17 OIR. For this purpose, we analyzed the protein expression of GFAP, as a glial activation marker, by Western blot and immunofluorescence in RA and OIR mice injected with NO_2_-OA or vehicle (Fig. 3A and C, respectively). Our results indicate that GFAP substantially increased at P17 OIR respect to RA retinas (Fig. 3A), and that increase was in accordance with activation of Müller glial cells which was corroborated by immunofluorescence staining (Fig. 3C). Fig. 3B showed the quantification of GFAP levels at P17 OIR relative to RA controls in presence or absence of NO_2_-OA treatment. Importantly, NO_2_-OA treatment did not change GFAP expression in RA mice (Fig. 3A and B). Moreover, we also observed low expression of glutamine synthase (GS) which suggest impairment in glutamate detoxification at P17 OIR with respect to RA retinas (Fig. 3A). The quantitative analysis indicated a significant decrease in GS levels in retina at P17 OIR mice, which was not modified by NO_2_-OA treatment (Fig. 3B). In summary, these results revealed that NO_2_-OA treatment attenuates gliosis, although NO_2_-OA does not improve glutamate detoxification by restoring GS levels at P17 OIR.

### 3.4 NO_2_-OA reduces the number of Iba1+ myeloid cells in OIR retinas

**Fig. 4:**
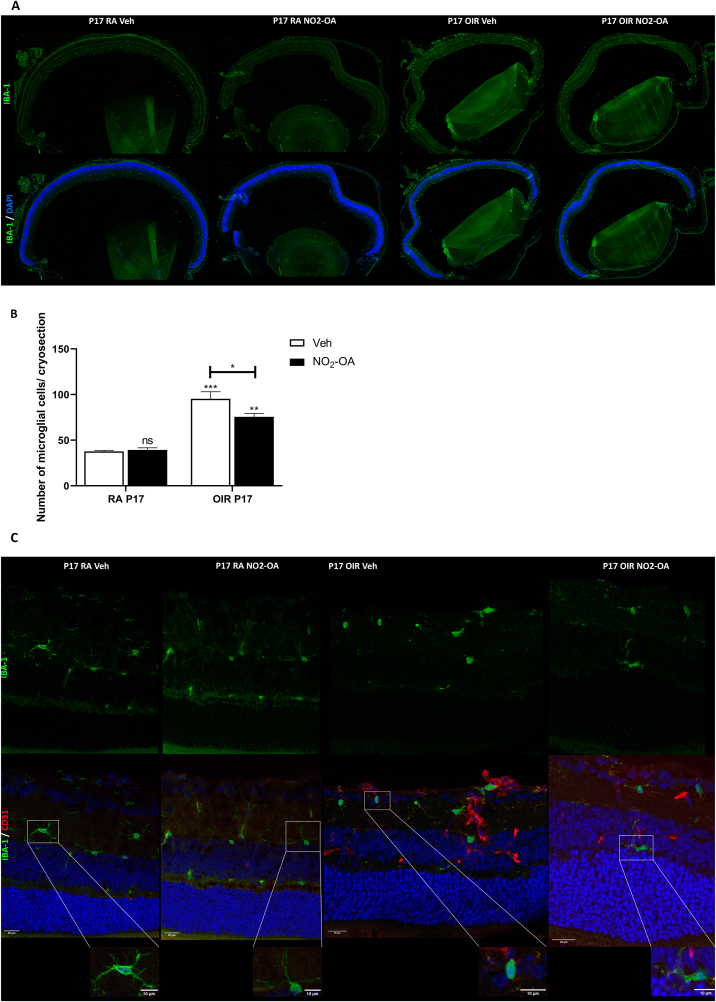
NO_2_-OA reduces the number of total IBA1+ myeloid cells in OIR retinas. **A)** Representative immunofluorescence analysis of IBA-1 (green) in cryosections of RA and OIR mouse retinas at P17 (upper panels). Cell nuclei were counterstained with Hoechst 33258 (blue) (lower panel). **B)** Quantification of microglial cells per cryosection. Data are presented as mean ± SEM. ns, non-significant, ∗p < 0.05, ∗∗p < 0.01, ∗∗∗p < 0.001. **C)** Representative immunofluorescence of microglial cell marker, IBA1 (green), in combination with an EC marker, CD31 (red). Cell nuclei were counterstained with Hoechst 33258 (blue). Scale bar: 20 μm.

IBA1+ myeloid cells activation in retina has been strongly linked to pathological retinal NV, increment of proinflammatory factor, tissue damage and neurodegeneration in PR [20,49,50]. Thus, we next evaluated the response of this cell population to NO_2_-OA treatment in the OIR model. For this purpose, cryosection of retinas at P17 from RA and OIR treated or not with NO_2_-OA were immuno-labeled for the ionized calcium binding adaptor molecule 1 (Iba1) as a marker of resident microglial cells and tissue infiltrating monocytes during inflammatory processes (Fig. 4A) [51]. A quantitative analysis in the whole retinal tissue showed a significant increase in the number of total Iba1+ cells in OIR compared to RA mice at P17. Interestingly, NO_2_-OA treatment at P17 OIR significantly reduced the number of Iba1+ cells (Fig. 4B). It has been demonstrated that density of Iba1+ cells increase in NV areas and they are aggregate and locate around the neovascular tufts on the vitreal surface of the retina [52]. Therefore, in order to study its localization and morphology, we perform co-immunostaining with Iba1 and anti-CD31 antibodies for endothelial cells (EC) [42]. As results, we observed an active Iba1+ cells with larger soma size and absent dendrites in the most highly vascularized area at P17 OIR, compared to the RA mice (Fig. 4C). NO_2_-OA-treated retinas displayed an increased in number of ramified Iba1+ cells or “resting” and reduced number of amoeboid Iba1+ cells with absent dendrites (activated cells) compared with OIR vehicle (Fig. 4C). These results suggest that NO_2_-OA has a beneficial effect in modulating the response of Iba1+ cells toward a resting phenotype in the OIR model.

### 3.5 NO_2_-OA improves retinal function and neurodegeneration

**Fig. 5:**
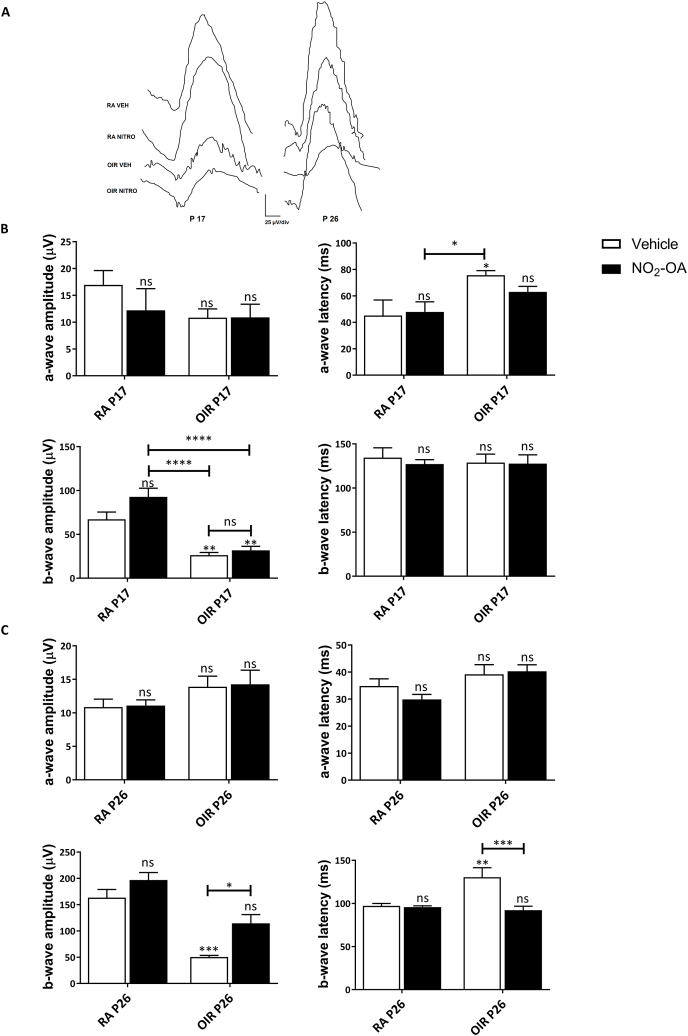
NO_2_-OA improves retinal function and neurodegeneration. **A)** Representative recordings of mixed response (scale 25 μV/div) at P17 and P26 OIR and RA mice. The light intensity used for ERG recordings was 10 cd s/m^2^. **B–C)** Amplitudes and latencies of a-waves and b-waves from scotopic ERG were recorded at P17 and P26 in OIR and RA mice. Data showed the average responses over both eyes, in seven mice per condition. Data are presented as mean ± SEM. ns, non-significant, ∗p < 0.05, ∗∗p < 0.01, ∗∗∗p < 0.001, ∗∗∗∗p < 0.0001.

In PR, vascular changes not only produce hypoxic avascular areas, but also increased vascular permeability leading to dysfunction and neuronal death [[8], [9], [10], [11]]. We have previously reported that OIR model at P17 alter the retinal function measured by scotopic ERG and that alteration persist until P26 despite vascular normalization [8,9]. Fig. 5 shows the amplitude and latency of ERG a- and b-waves at P17 and P26 of RA and OIR mice treated or untreated with NO_2_-OA. The b-wave amplitude (mainly INL cell response) was significantly reduced in OIR model, which may be attributed to synaptic contacts loss and even neuronal death in the inner retina (Fig. 5A). However, NO_2_-OA treatment on OIR model showed a tendency to recover b-wave amplitude at P17, although this recovery becomes significant at P26 (Fig. 5B and C). As expected, a-wave amplitude (mainly ONL cell response) at P17 and P26 was not altered in the OIR model [38]. Also, a tendency toward larger latencies was observed in P17 OIR mice with respect to RA (Fig. 5B), and b-wave latency increase at P26 OIR was indicative of progressive retinal dysfunction (Fig. 5C). Regarding the a-wave latency, it was significantly increased at P17 OIR with respect to RA mice, condition that was reverted at P26 (Fig. 5B–C). In addition, NO_2_-OA treatment decreases b-wave latency to control levels at P26 OIR. Thus, scotopic ERG response suggests that NO_2_-OA treatment prevents loss of retinal function in OIR mouse model.

### 3.6 NO_2_-OA ameliorates cell death at P26 OIR mice

**Fig. 6:**
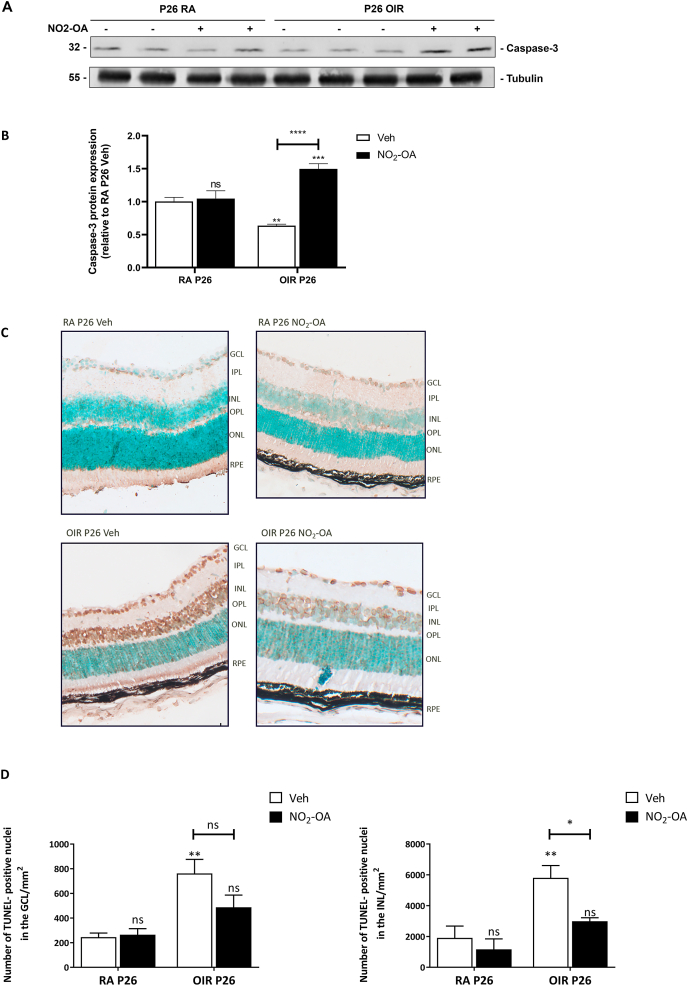
NO_2_-OA ameliorates cell death at P26 OIR retinas. **A)** Representative Western blot of caspase-3 from retinas of RA and OIR mice at P26 treated or not with NO_2_-OA. Tubulin is shown as a loading control. **B)** Quantification of Caspase-3 was performed by densitometry and normalized to Tubulin. **C)** Representative TUNEL cryosections of RA and OIR retina at P26 treated or not treated with NO_2_-OA. Abbreviations: GCL, ganglion cell layer; IPL, inner plexiform layer; INL, inner nuclear layer; OPL, outer plexiform layer; ONL, outer nuclear layer; RPE, retinal pigment epithelium. Arrows are indicating TUNEL-positive nuclei. **D)** Quantification of the TUNEL-positive cells in GCL and INL at P26 are shown. Data are presented as mean ± SEM. ns, non-significant, ∗p < 0.05, ∗∗p < 0.01, ∗∗∗p < 0.001, ∗∗∗∗p < 0.0001.

Since NO_2_-OA exhibited protective effect on retinal dysfunction in the OIR model, our next goal was to explore caspase-3 levels as a marker of cell death (Fig. 6A). Our result demonstrates a significant decrease of protein expression of total caspase-3 in retina at P26 OIR (Fig. 6B), suggesting that caspase-3 may be cleaved to small forms. Interestingly, NO_2_-OA treatment prevented the decrease of caspase-3 at P26 OIR (Fig. 6B). Thus, we evaluated neuronal death in retinal cryosections at P26 OIR by TUNEL assay. TUNEL-positive nuclei were detected in the ganglion cell layer (GCL), in the inner nuclear layer (INL; mainly neurons such as bipolar and amacrine cells), and in few cells, into the outer nuclear layer (ONL; photoreceptors) in OIR retinas (Fig. 6C). Quantification of TUNEL-positive cells showed that OIR is significantly higher than RA control mice, and NO_2_-OA treatment was able to decrease or prevent neuronal death in OIR (Fig. 6D). These results suggest that NO_2_-OA has beneficial effects on retinal cells, since it improves the functionality and survival of neurons from GCL and INL at P26 OIR retinas.

### 3.7 NO_2_-OA attenuates ROS induced by aqueous humor from patients with PR in MGCs

**Fig. 7:**
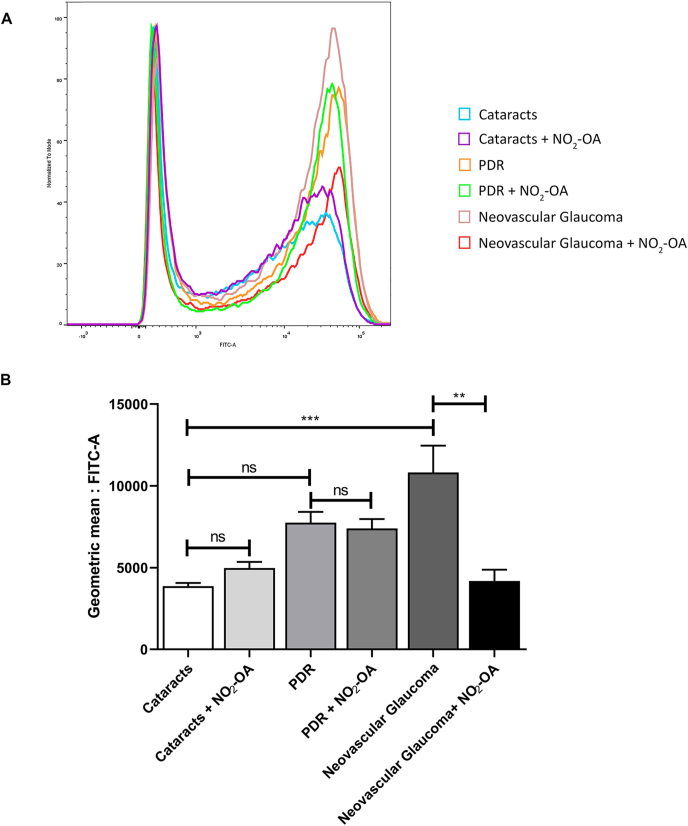
NO_2_-OA attenuates ROS induced by aqueous humor from patients with PR in MGCs. **A)** Representative histogram of ROS. ROS levels were determined with a DCF-DA probe using flow cytometry. **B)** Quantification data are presented as means ± SEM of the geometric mean and were analyzed using a one-way ANOVA followed by Tukey's post-test. ns, non-significant, ∗∗p < 0.01, ∗∗∗p < 0.001.

Previous studies have demonstrated that oxidative stress, inflammation and vascular injury are involved in the pathogenesis and progression of PR [53,54]. One of the first signs of inflammation in PR is the activation of MGCs and microglia. With this in mind, we collected aqueous humor from diabetic patients with different stages of progression of PR (PDR and neovascular glaucoma) that course with glial activation [[55], [56], [57]]. As controls, we included non-diabetic patients with absence of PR. Next, we evaluated if aqueous humor collected from patients with PR induce activation and increase of oxidative response in MIO-M1 cells. For this purpose, we measured ROS using the DCF-DA probe by flow cytometry in MIO-M1 cells [43] incubated in presence or absence of NO_2_-OA (5 μM) prior to stimulation with aqueous humor with the aforementioned conditions (Fig. 7A). Aqueous humor from patients with PDR and neovascular glaucoma significantly increased ROS levels in MIO-M1 cells compared to patients with cataracts (control). However, pretreatment of MIO-M1 cells with NO_2_-OA inhibit ROS increase induced by aqueous humor from patient with neovascular glaucoma (Fig. 7B). Overall, these results demonstrate the protective effect of NO_2_-OA against the oxidative response induced by pathological aqueous humor in MGCs.

## 4 Discussion

Pathological NV in the retina is a leading cause of partial vision loss that gradually progress to blindness in PDR and ROP [2,3]. Accumulating evidence, stemming from experimental models and clinical studies of PR has provided insights into the mechanisms of vascular injury. However, it is known that non-vascular cells such as glia (MGCs and microglial cells) and neurons are also affected with the consequent production of gliosis, activation and increase of microglia number, and neurodegeneration (retinal functional loss, altered neurotrophic balance and neuronal damage) [8,9,20,50]. Previous research has indicated that an optimal therapeutic strategy for ischemic retinopathies would ideally involve restore normal vascular function in the remaining vasculature and mitigate or prevent neurodegenerative changes in the retinal tissue [58].

Among the etiopathogenic mechanisms of PR converge hypoxia, inflammation, oxidative and nitrosative damage [53,59]. In fact, several studies have been carried out to reduce the excessive accumulation of ROS or reduce ROS production in retinopathies [[60], [61], [62]]. One beneficial therapeutic strategy explored in ROP and PDR is the modulation of the antioxidant pathway, Keap1/Nrf2, which regulates the expression of phase II antioxidant enzymes such as heme oxygenase-1 (HO-1), NAD(P)H quinone oxidoreductase 1 (NQO1), as well as enzymes involved in glutathione biosynthesis, among others [22,[25], [26], [27],^63^]. Furthermore, it is known that the activation of the Keap1/Nrf2 pathway in the OIR model decreased the avascular area and pathologic NV at P17 [26,27,64]. Recently, numerous studies have demonstrated the anti-inflammatory and cytoprotective effect of nitro-fatty acids (NO_2_-FA) including nitro-oleic acid (NO_2_-OA), and conjugated nitro-linoleic acid (NO_2_-CLA) in different experimental models [[65], [66], [67]]. Accordingly, we previously demonstrated, *in vitro*, the protective effect of NO_2_-OA on oxidative stress, gliosis, and pro-angiogenic response in MGCs [13]. Herein, we investigated the effects of NO_2_-OA on neovascularization, gliosis, microglia activation and neurodegeneration in the OIR mouse model.

First of all, we investigate the response of Keap1/Nrf2 pathway to NO_2_-OA in healthy retina [[25], [26], [27],^64^]. Thus, we demonstrate that a single i.o. injection of NO_2_-OA was able to induce the transcriptional expression of Nrf2 downstream genes, NQO1 and SOD1, in healthy retina. Our results showed a dose-dependent increase in NQO1 mRNA expression in adults’ mice (Fig. 1A). Additionally, evaluation of systemic administration via i.p. injection of NO_2_-OA, at P12, also showed a significantly increased in NQO1 and SOD1 expression in the retina (Fig. 1B). Similar effect on antioxidant enzymes has been reported with pharmacological activators of Nrf2 (RS9, dh404 and CDDO-imidazolide), post-injection in the retina [23,26,27]. Nrf2 activation is the target to alleviate pathological conditions associated with neurodegenerative disorders. In fact, in angiogenesis has been described a dual role for Nrf2 pathway as a positive and negative regulator. Therefore, Wei et al. [25] described that Nrf2-deficient mice exhibit an exacerbation of NOX2 in OIR model responsible of the adverse effects for reparative angiogenesis, a biological process necessary for ischemic revascularization. In another *in vivo* experiment, the injection of Nrf2 activator RS9 inhibited NV and increases the expression of tight junction proteins decreasing the vascular leakage in the retina and supporting the beneficial action of Nrf2 activation [27].

Since we previously showed that NO_2_-OA increases the expression of Nrf2 target genes without affecting viability in the MGCs [13], we assessed NO_2_-OA toxicity in healthy retina in mice. NO_2_-OA administration has not induced harmful changes in retinal function and histology as shown in Fig. 1C–E. The retina contains a high concentration of unsaturated fatty acid, especially docosahexaenoic acid (DHA), which is essential for the normal functioning of the retina and high levels of DHA has been associated with decrease risk to develop retinal disease such as DR or glaucoma [68,69]. Although we have not measured the endogenous formation of nitro-fatty acids (NO_2_-FA) in the retina, these have been found in the plasma and urine of healthy individuals, with conjugated nitro-linoleic acid (NO_2_-CLA) being the most representative form. In this work, we have explored the health benefit of NO_2_-OA in the retina, but future studies will be necessary to elucidated the function of the MGCs as potential mediator of nitration of conjugated fatty acid at local retina.

The OIR is a well-established mouse model that mimics the findings described in ROP and the proliferative stage of DR, working as a robust tool to investigate underlying mechanisms and develop therapeutic strategies [36]. Here, we observed a peak of NV and vaso-obliteration, gliosis, microglial activation, a decrease in the levels of GS and neurodegeneration at P17 OIR. Furthermore, when the vascular alterations were resolved at P26 OIR, the neurodegeneration persisted, in accordance with other studies [8,9,36]. Initially, we evaluated the NO_2_-OA effect on NV and vaso-obliteration at P17 OIR. NO_2_-OA treatment induced normal and functional reparative angiogenesis, reducing both the avascular and neovascular retinal areas (Fig. 2B and C). These results support previous works demonstrating that pharmacological activators of Nrf2, such as dh404 and RS9, decreased avascular and neovascular areas and improved the vascular permeability in the retinas of OIR model [26,27]. Moreover, genetic ablation of Nrf2 (Nrf2^−/−^) in P17 OIR mice significantly increased the avascular area and pathological preretinal NV [24,25].

Since pathological NV is mainly regulated by excessive VEGF production in hypoxic cells of retinal tissue, we evaluated VEGF mRNA expression in OIR and RA retinas at P17 to elucidate the role of NO_2_-OA in reparative angiogenesis. As expected, our results showed an increase in VEGF expression levels in retinas at P17 OIR compared to RA mice; however, NO_2_-OA injection did not modify VEGF expression in the retina (Fig. 2D). This result may have two interpretations, one of them is that NO_2_-OA not induce changes in VEGF expression and the second one may be related to a dilution effect when analyzing VEGF expression in the whole retina. Additionally, NO_2_-OA treatment did not change PEDF mRNA expression (Fig. 2E). Similar results were described in experiments performed in bovine aortic endothelial cells (BAEC) demonstrating a direct anti-angiogenic effect of NO_2_-OA in tubulogenesis assays [13]. Therefore, we speculate that modulation of NV is likely due to direct effect of NO_2_-OA on ECs and independent of VEGF or PEDF modulation.

Interestingly, NO_2_-OA attenuates GFAP expression in activated MGCs when retinas were under hypoxic conditions in the OIR model (Fig. 3A and B) [8,9]. This effect helps to avoid the establishment of a chronic gliosis and reduce the progressive deleterious response of MGCs that could damage retinal tissue. On the other hand, as previously described a prevalent function of MGC is to regulate glutamate levels through GS, a key neuroprotective enzyme that converts glutamate into glutamine. Here, GS expression decreased in retinas at P17 OIR but, unlike GFAP, NO_2_-OA did not restore GS expression (Fig. 3A) [70].

In the OIR model, it has been reported that the number of total IBA1+ retinal myeloid cells increase in response to both hyperoxia and hypoxia stimulus [16,20,50,61], with predominant localization in areas of ischemia and surrounding neovessels [71]. We have described similar findings in this report (Fig. 4A and B). In fact, it has been previously showed migration of IBA1+ cells from the outer towards the inner retina, as well that resident microglia proliferate and constitute the predominant myeloid cell population in areas of ischemia and NV, while the number of blood-derived Mϕ is substantially lower [71]. These observations strongly indicated a local proliferation and migratory redistribution of microglial cells at P17 OIR. We also found that during NV peak, IBA1+ cells remained activated in OIR mice displaying an amoeboid and less-ramified morphology (Fig. 4C). However, when we treated with NO_2_-OA a significant reduction in the number of IBA1+ cells were observed at OIR P17. These cells were characterized by a small cell body and highly ramified processes, indicating a “resting” state and possibly pro-regenerative, which may be related with the anti-inflammatory effect of NO_2_-OA.

In the PR, glial cells, including MGCs and microglia, remained activated and secrete several pro-inflammatory cytokines, such as TNF-α, IL1-β, IL-6, which participate in pathological angiogenesis [53,60]. While measuring cytokine levels during the OIR model and in response to NO_2_-OA could be of relevance and necessary to demonstrate their anti-inflammatory activity, potential changes in the kinetics of cytokines expression have yet to be further investigated. Anyway, NO_2_-OA anti-inflammatory action has been described through inhibition of NF-κB signaling, assembly of TLR4 and inhibition of inflammasome, affecting monocytes, macrophages and ECs [72,73].

Interestingly, we observed an altered retinal function measured by ERG. The b-wave amplitude and b-wave latency were altered at P17 and P26 OIR with respect to RA mice (Fig. 5A–C). This progressive retinal dysfunction may be attributed to losing synaptic contacts and even neuronal death in the inner retina. Many studies have, previously, reported similar alterations in OIR retinas [8,9]. NO_2_-OA showed a trend toward restoring b-wave amplitude and latency at P17, which was significant at P26 OIR mice (Fig. 5A–C). The beneficial changes with NO_2_-OA treatment at P17, seems to play a positive role improving retinal function at P26 OIR. These results suggest a protective effect of NO_2_-OA against the neurodegeneration in the OIR model. To further corroborate these findings, we analyzed total caspase-3 levels and cell death at P26 OIR. A significant decrease in total caspase-3 was observed, indicating cleavage of caspase-3 into its active form and suggesting activation of apoptosis (Fig. 6A and B). Furthermore, in line with these results we observed a significant increase in the cell death in the INL and GCL by TUNEL assay at P26 OIR (Fig. 6C and D). Both effects were prevented by NO_2_-OA treatment (Fig. 6A–D). These findings undoubtedly invite further investigation into the mechanism by which NO_2_-OA exerts a neuroprotective effect in the OIR model.

Previous observations have shown that levels of cytokines measured in the aqueous humor of patients with diabetic eye disease, reflect the levels measured in the vitreous of these patients [74]. In addition, we have demonstrated, *in vitro,* the induction of ROS and gliosis in MGCs exposed to different stimulus that mimic retinopathies [13]. Motivated by these findings, we next examined if the NO_2_-OA treatment was able to modulate ROS induced in MGCs by aqueous humor from patients with different stages of PR. Here, we found that NO_2_-OA significantly decreased ROS levels induced in MGCs by aqueous humor of patients with neovascular glaucoma. Since the highest ROS levels were observed in the aqueous humor of neovascular glaucoma respect to PDR patients and considering these samples represent the end-stage of proliferative diabetic retinopathy, their effect could be associated to the aggressiveness of neovascular proliferation.

In summary, in this study we demonstrated the diverse actions of NO_2_-OA in the time course of alterations observed in the mouse model of OIR. Interestingly, NO_2_-OA was able to attenuate retinal vaso-obliteration and NV, promote reparative retinal angiogenesis, and rescue neurons and glial cells from death. In this regard, the finding that NO_2_-OA has a beneficial effect on retinal tissue constitutes the first evidence that nitro-fatty acids may emerge as a potential therapeutic agent in preventing complex proliferative and neurodegenerative disorders like ROP and PDR.

## Funding

This work was funded by grants from Secretaría de Ciencia y Tecnología, Universidad Nacional de Córdoba (SECyT-10.13039/100010442UNC) PIDTA 2023 (RESOL 2024-21-E-UNC-SECYT#ACTIP), Fondo para la Investigación Científica y Tecnológica (FONCyT), Proyecto de Investigación en Ciencia y Tecnología (PICT) 2020 N^o^ 01586, and PIP (CONICET) 11220200100830CO (all to M.C.S.). Secretaría de Ciencia y Tecnología, Universidad Nacional de Córdoba: Consolidar-2018 (RESOL 411-2018-UNC-SECYT), PIDTA-2023 (RESOL 2024-21-E-UNC-SECYT#ACTIP) and FONCyT: Préstamo BID-PICT 2019 N^o^ 2083 (all to G.B.).

## CRediT authorship contribution statement

**María Victoria Vaglienti:** Writing – review & editing, Writing – original draft, Validation, Methodology, Investigation, Formal analysis, Data curation, Conceptualization. **María Constanza Paz:** Methodology, Investigation, Formal analysis. **María Victoria Gutierrez:** Methodology, Investigation, Formal analysis. **Paula Virginia Subirada:** Methodology, Investigation, Formal analysis. **Jose Luna:** Investigation, Conceptualization. **Gustavo Bonacci:** Writing – review & editing, Writing – original draft, Supervision, Resources, Project administration, Investigation, Funding acquisition, Conceptualization. **María Cecilia Sánchez:** Writing – review & editing, Writing – original draft, Supervision, Resources, Project administration, Investigation, Funding acquisition, Conceptualization.

## Declaration of competing interest

The authors declare no conflict of interest.

## Data Availability

Data will be made available on request.
