# Corrigendum to “Nitro-Oleic acid protects from neovascularization, oxidative stress, gliosis and neurodegeneration in oxygen-induced retinopathy” [Redox Biol. 83 (2025) 103634]

**DOI:** 10.1016/j.redox.2025.103725

**Published:** 2025-06-14

**Authors:** María Victoria Vaglienti, María Constanza Paz, María Victoria Gutierrez, Paula Virginia Subirada, Jose Luna, Gustavo Bonacci, María Cecilia Sánchez

**Affiliations:** a Departamento de Bioquímica Clínica, Facultad de Ciencias Químicas, Universidad Nacional de Córdoba, Córdoba, 5000, Argentina; b Centro de Investigaciones en Bioquímica Clínica e Inmunología (CIBICI), Consejo Nacional de Investigaciones Científicas y Técnicas (CONICET), Córdoba, 5000, Argentina; c Centro Privado de Ojos Romagosa S.A, Argentina

The authors regret that the first names and given names were interchanged in the originally published version. The correct presentation of the author names is provided below.

María Victoria Vaglienti, María Constanza Paz, María Victoria Gutierrez, Paula Virginia Subirada, Jose Luna, Gustavo Bonacci, María Cecilia Sánchez.

The author names have been corrected in the originally published version. The authors would like to apologise for any inconvenience caused.

